# Complete Hydatidiform Mole Presenting as a Placenta Accreta in a Twin Pregnancy with a Coexisting Normal Fetus: Case Report

**DOI:** 10.1155/2012/405085

**Published:** 2012-08-13

**Authors:** Marijo Aguilera, Philip Rauk, Rahel Ghebre, Kirk Ramin

**Affiliations:** ^1^Division of Maternal-Fetal Medicine, Department of Obstetrics and Gynecology, University of Minnesota, Minneapolis, MN 55454, USA; ^2^Division of Gynecologic Oncology, Department of Obstetrics and Gynecology, University of Minnesota, Minneapolis, MN 55454, USA

## Abstract

A twin pregnancy with a complete hydatidiform mole and a coexisting normal fetus (CHMF) is a rare clinical scenario, and it carries many associated pregnancy and postnatal risks. Limited numbers of case studies exist reporting an outcome of live birth, and only three prior cases report the presentation of a hydatidiform mole as a placenta previa. We report a case of CHMF with the molar component presenting antenatally as a placenta previa, which ultimately resulted in placenta accreta at the time of delivery. A live male infant was delivered at 34 weeks' gestation via planned cesarean section, and a hysterectomy was performed following unsuccessful removal of the molar component. We additionally utilized previously described methods of placing internal iliac balloons and ureteral stents prior to delivery. In such a high-risk pregnancy with a known molar previa component, these surgical preparation measures may be of benefit.

## 1. Introduction

Twin gestations comprising a complete hydatidiform mole and a coexisting normal fetus (CHMF) are rare occurrences, with an estimated incidence of 1 in 22,000–100,000 [1]. In many instances, these pregnancies are associated with significant maternal and fetal complications including preeclampsia, thromboembolic disease, hyperemesis, hemorrhage, and intrauterine demise. Additionally, patients with CHMF may have an increased risk of persistent trophoblastic disease, although others identify this risk as similar to that after a singleton molar pregnancy [2]. We report a case of CHMF with the molar gestation presenting as a placenta previa. At the time of scheduled cesarean section at 34 weeks' gestation, the additional clinical diagnosis of a placenta accreta was made, resulting in a cesarean-hysterectomy. A multidisciplinary approach to the delivery with placement of bilateral internal iliac balloons and bilateral ureteral stents minimized maternal morbidity and resulted in the safe delivery of viable male infant.

## 2. Case Report

**Figure 1:**
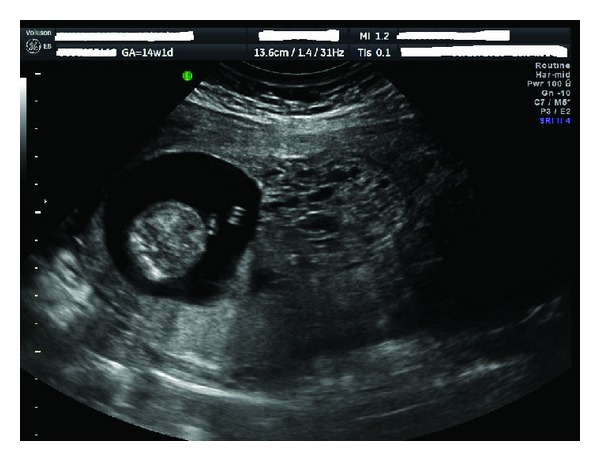
Ultrasound image of twin pregnancy with molar component presenting as a placenta previa and coexisting normal twin at 14 + 1 weeks' gestation.

A 48-year-old, gravida 1, para 0 female was referred to the University of Minnesota Maternal-Fetal Medicine Center due to vaginal bleeding and uterine fibroids. Ultrasound findings revealed a viable gestation at 14 + 1 weeks' and an adjacent thickened, nonhomogeneous placenta with a diffuse multicystic appearance (see Figure 1). The adjacent placental mass was consistent with a coexisting molar gestation, and it was noted to be overlying the cervix. In addition, there were multiple intramural uterine myomas visualized, the largest of which measured 10.3 cm × 9.3 cm × 7.3 cm and 7.0 cm × 7.1 cm × 6.2 cm. A serum quantitative hCG was 290,000 iu/L. The patient reported persistent vaginal bleeding starting early in the first trimester, not associated with cramping. Given the diagnosis of a presumed molar gestation, the patient was counseled on the risks of continuing the pregnancy. An amniocentesis was performed at 16 weeks' gestation which returned as a normal 46, XY karyotype. Amniotic fluid AFP was 1.38 multiples of the median. After identifying a viable fetus, the parents opted to continue the pregnancy. Further evaluation included normal thyroid function tests, and in agreement with the patient, we proceeded with imaging studies, as the presence of any metastases may have resulted in her decision to terminate the pregnancy. MRI of the brain, abdomen, and pelvis were negative for evidence of metastatic choriocarcinoma, and the molar pregnancy measured 7.0 cm × 8.3 cm × 6.9 cm. We thoroughly reviewed the extremely low teratogenic and stochastic risks of radiation from a chest CT (<1 rad) prior to obtaining a pulmonary CT scan that revealed small incidental nodules not concerning for metastases.

At 17 weeks, the patient was admitted to the hospital for observation due to an increase in vaginal bleeding. Her hemoglobin was stable at 11.6 g/dL, and the remainder of her laboratory analysis was reassuring. She was discharged to home on modified bedrest. 

A comprehensive ultrasound exam at 18 weeks' gestation demonstrated a normal live male fetus without evidence of anomalies. The coexisting molar pregnancy was confirmed to be covering the internal cervical os as a complete placenta previa. A repeat quantitative hCG was 208,900 iu/L.

At 20 weeks, the patient again presented to the hospital with a marked increase in vaginal bleeding and passage of large clots. Her labs remained stable with a hemoglobin of 11.0 g/dL, and her hCG had decreased to 159,690 iu/L. The decision was made to hospitalize the patient for the remainder of her pregnancy due to the persistent vaginal bleeding in a complete previa molar pregnancy. Her antenatal care included the maintenance of a current type and screen, monitoring hemoglobin levels, and serial hCG levels. The patient continued to have intermittent spotting throughout the duration of her pregnancy, but her hemoglobin remained in the normal range. Her hCG level declined progressively to a level of 44,506 at 32 weeks' gestation. Successive ultrasound examinations confirmed appropriate fetal growth, amniotic fluid, and umbilical artery doppler studies. The molar pregnancy remained a complete previa, and measured 10 cm × 16 cm × 8 cm at her 30 week ultrasound exam. The patient also met with the gynecologic oncology service, who counseled her on the risk for persistent gestational trophoblastic disease, and established a plan for repeat imaging should the hCG levels plateau or increase.

Her pregnancy was further complicated by gestational diabetes requiring glyburide as well as mild preeclampsia which she developed at 28 weeks' gestation. Her blood glucose levels and blood pressures were followed closely, and serial HELLP labs were within normal limits. She did not develop symptoms associated with severe preeclampsia. She received betamethasone injections at 23 weeks followed by a rescue course at 28 weeks. Antenatal testing included twice weekly biophysical profiles initiated at 32 weeks and twice daily nonstress tests since admission. We did not initiate prophylactic anticoagulation during her prolonged hospitalization as this is not standard policy in our institution; in addition we thought it not wise in light of her persistent bleeding and risk of hemorrhage.

A comprehensive delivery plan was established and discussed with the parents. A decision was made to proceed with planned cesarean section at 34 weeks' gestation. In view of the concern for excessive bleeding secondary to the presence of multiple uterine fibroids and the molar pregnancy presenting as a complete previa, prophylactic control of local vascular flow was established by placing bilateral internal iliac artery balloon catheters, performed by interventional radiology (IR). Additionally, bilateral ureteral stents were placed prior to initiating the procedure. Both procedures were performed in the operating room under spinal anesthesia; urology placed stents via cystoscopy and IR gained access to the common femoral arteries under direct ultrasound visualization, followed by placement of a 7 French vascular sheath guided over the aortic arch to the internal iliac arteries using fluoroscopy. A 14 mm × 2 cm balloon was then inserted on each side. Balloon inflation to 2 atmospheres demonstrated appropriate arterial occlusion.

**Figure 2:**
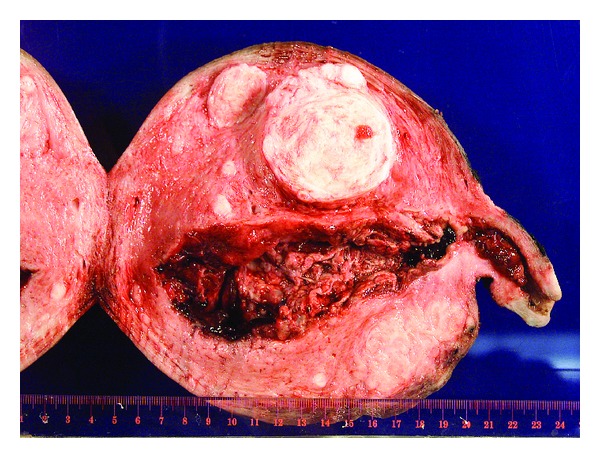
Pathology specimen of entire gross uterus and molar component within the lower uterine segment extending through the internal cervical os. Normal placental bed visualized within the fundal portion of the endometrial cavity. Multiple fibroids seen within the uterus.

General anesthesia was then administered and a classical cesarean section was performed via vertical skin incision. A viable male infant was delivered with a weight of 2310 g and apgars of 7 and 9 at 1 and 5 minutes, respectively. The placenta of the live fetus was extracted easily. The classic hydropic villi of the molar component appeared to be degenerating, and were very adherent to the endometrium, clinically consistent with a placenta accreta. At this point there was significant uterine bleeding, thus a cesarean hysterectomy was initated with the molar pregnancy in situ. The internal iliac arterial balloons were inflated and a total hysterectomy was performed without complications. There was minimal bleeding during the hysterectomy, and estimated blood loss was 1000 mL for all procedures combined. Total time of arterial balloon inflation was 30 minutes, as they were deflated following ligation of the uterine arteries. The patient's postoperative course was unremarkable and she was discharged home on postoperative day number 6. Pathological examination of the second twin/placental mass confirmed a molar pregnancy (see Figure 2). Given the markedly advanced degeneration of the molar tissue and familiarity with identifying molar tissue as invasive in regards to a neoplastic process only, the pathologist had difficulty confirming an accreta on examination.

Weekly hCG levels were followed after delivery, and at 6 weeks postpartum, they had reached a plateau. An hCG tumor marker assay remained in the 40s. A computed tomography (CT) scan was obtained and demonstrated no evidence of disease. However, given 4 values of hCG levels at a plateau, it was decided to treat her persistent gestational trophoblastic disease. Work up for metastatic disease including repeat CT imaging at this point was negative. Methotrexate administration was administered at 50 mg/m^2^, and hCG levels fell to <3 IU/L after 4 weekly injections. Her hCG levels have remained within the normal level, continuing followup for one year.

## 3. Discussion

Twin gestations consisting of a complete hydatidiform mole and a coexisting normal fetus (CHMF) are rare, with an estimated incidence of 1 in 22,000–100,000 [1, 2]. There are multiple case reports and series of CHMF documented; these pregnancies are often associated with severe maternal complications such as persistent vaginal bleeding, thromboembolic disease, severe preeclampsia, and persistent gestational trophoblastic neoplasia (GTN) [1–12]. Additionally, the largest case series identifying 53 continuing pregnancies reported fetal complications including spontaneous abortion or intrauterine death of the normal fetus in approximately 60% of cases [3]. However, among the nearly 40% live births in this series, the outcome was favorable, with the median gestational age of delivery at 35 weeks [3]. Furthermore, the rate of persistent GTN requiring chemotherapy in women electing to continue with pregnancy was not different from patients who either had a singleton molar pregnancy, or CHMF pregnancies who decided to terminate [3]. This data is in contrast to previous smaller series of CHMF which indicate a risk of persistent GTN of approximately 50% [4, 5].

Other authors have concluded that persistent GTN occurs more frequently in CHMF gestations delivering prior to viability versus those that result in a live fetus (68.4% versus 28.6%) [13]. The CHMF pregnancies that resulted in a live fetus were also associated with significantly lower predelivery serum hCG levels than those ended prior to viability (167, 883 mIU/mL versus 1,078,417 mIU/mL). This suggests either a less aggressive or a degenerating gestational trophoblastic disease [13]. In our case, the hCG levels peaked at 290,000 and declined to 44,506 at 32 weeks' gestation, consistent with a degenerating trophoblastic disease. This may be a good prognostic sign in the case of persistent GTD. 

There is a lack of an accurate estimate of persistent GTN in CHMF gestations. Approximately 30 cases of CHMF gestations have documented live birth of a viable second twin. These increasing reports put into question the historical recommendations to terminate CHMF pregnancies. Additionally, although some authors have argued that the potential of developing persistent GTD in women with CHMF pregnancies is higher, advanced gestational age does not appear to be a risk factor [4]. This suggests that continuing pregnancy and achieving a successful live birth may be an option for patients who clearly understand the increased aforementioned risks [1–5].

Among reported CHMF pregnancies, there have been three cases of the hydatidiform mole presenting as a placenta previa. Ongura et al. described a 30-year-old primigravida who presented at 20 weeks' gestation with vaginal bleeding. Ultrasound findings were suggestive of a molar pregnancy. The patient was ultimately delivered via hysterotomy 1 week after admission due to massive bleeding. Placental pathology confirmed a complete mole [5]. Suri et al reported a case of CHMF complicated by placenta previa that subsequently developed an intrauterine infection and abscess formation within the molar pregnancy tissue. The patient exhibited signs of systemic inflammatory response at 28 weeks, thus the decision was made to proceed with delivery. A live male infant was delivered via hysterotomy and the infected molar pregnancy was removed with pathology showing a classic mole and bacterial abscess [14]. Finally, Klatt et al. reported a case of a complete molar pregnancy that extended over the internal cervical os as a placenta previa. The patient was admitted to the hospital at 26 weeks due to persistent spotting, and was subsequently delivered at 31 weeks due to increased bleeding and fetal heart rate changes. A live male infant was delivered via classical hysterotomy, followed by spontaneous separation of the molar gestation. Uterine artery balloons were inflated prior to the cesarean section as a prophylactic measure because of the increased risk of hemorrhage [15].

Although these examples highlight molar pregnancies presenting as a placenta previa, our case is the first to report a molar pregnancy complicated by placenta previa with a concomitant clinical diagnosis of placenta accreta, ultimately resulting in hysterectomy. As suggested by Klatt et al., persistent vaginal bleeding may have been due to the previa versus an aggressive molar component, since in both cases, hCG levels were on the lower end (200,000 mIU/mL in the case by Klatt and 290,000 in our case). Our documentation of the sonographic appearance was also in agreement with Klatt's history of the molar component in that it demonstrated an increase in size throughout gestation, likely due to hemorrhage [15]. In retrospect these sonographic findings may have also been clues to a possible diagnosis of an accreta due to the degenerative and subsequent adherent nature of the molar tissue.

We also incorporated the use of internal iliac artery balloon occlusion and ureteral stent placement to assist in this complicated surgical management of other “nonmolar” placenta accretas. It is known that placenta previa is associated with an increased risk of placenta accreta (approximately 3.3% in a first cesarean section) [16]. Additional risk factors for placenta accreta present in our patient include advancing maternal age and uterine leiomyomatas [16]. Furthermore, molar pregnancies alone are associated with a significant risk of hemorrhage at the time of evacuation, thus it seems prudent to employ prophylactic measures in attempt to minimize surgical blood loss. Although our antepartum sonographic evaluations did not suggest the presence of a placenta accreta, given her above risk factors, anticipation of a cesarean hysterectomy allowed for reduction of maternal morbidity. Perhaps the major component of preparation for such a procedure includes recognizing that placenta accreta may lead to massive obstetric hemorrhage. Thus we chose to proceed with temporary occlusion of the internal iliac arteries, which has been advocated by some authors as a method of decreasing blood loss [17, 18].

In summary, pregnancies complicated by CHMF may result in a viable liveborn infant approximately 40% of the time [3]. Continuing such a pregnancy may be an option, granted the mother has been appropriately counseled on the numerous risks. In cases where the molar component is presenting as a placenta previa, the degenerating molar tissue may further clinically manifest as a placenta accreta. Thus, anticipation for increased surgical blood loss and possible cesarean hysterectomy may assist in decreasing maternal morbidity and mortality. A complete multidisciplinary approach to assist in the management of these difficult surgical cases has become increasingly more common, and our case highlights the benefits of such a team approach.
